# Kyste épidermoide de la selle turcique: à propos d’un cas

**DOI:** 10.11604/pamj.2017.28.39.10502

**Published:** 2017-09-15

**Authors:** Ghizlane El Mghari, Bouchra Rafiq, Nawal El Ansari

**Affiliations:** 1 Service d’Endocrinologie, Diabétologie et Maladies Métaboliques, Hôpital Arrazi, CHU Mohammed VI, Laboratoire de Recherche de Pneumo-Cardio-Immunopathologie et Métabolisme (PCIM), Faculté de Médecine et de Pharmacie de Marrakech, Université Cadi Ayad, Marrakech, Maroc

**Keywords:** Kyste épidermoide, congénitale, antéhypophysaire, Epidermoid cyst, congenital, anterior pituitary

## Abstract

Le kyste épidermoïde (KE) ou cholestéatome est une tumeur bénigne d'origine souvent congénitale, survenant dans les espaces sous-arachnoïdiens, son traitement est principalement chirurgical. Nous rapportons le cas d'un patient, âgé de 38 ans, qui a présenté d'une façon progressive, un syndrome d'hypertension intracrânienne, associé à des signes d'insuffisance antéhypophysaire des axe corticotrope, thyréotrope et gonadotrope installés depuis 1an et chez qui l'IRM cérébrale a été en faveur d'un craniopharyngiome, avec révélation de kyste épidermoide sur pièce anatomo-pathologique.

## Abstract (en)

Epidermoid cyst (EC) or cholesteatoma is a benign tumor, often of congenital origin, occurring in the sub-arachnoidian spaces. Its treatment is based on surgery. We report the case of a 38 year-old patient presenting with progressive intracranial hypertension syndrome associated with anterior pituitary insufficiency signs of the corticotropic, thyrotropic, gonadotropic axis occurring 1 year before. Brain MRI showed a craniopharyngioma. Anatomopathologic evaluation of the specimen showed epidermoid cyst.

## Introduction

Le kyste épidermoïde (KE) ou cholestéatome est une tumeur bénigne d'origine souvent congénitale, il représente 1% des tumeurs intracrâniennes et se localise essentiellement au niveau de l'angle ponto-cérébelleux et en suprasellaire [1]. L'IRM en séquence pondérées en T1 et en T2, montre une lésion de signal proche de celui du LCS mais souvent hétérogène notamment en T2 en iso-ou hyposignal T1, hypersignal T2. Sur pièce anatomopathologique le KE prend l'aspect d'une tumeur perlée avec une surface nodulaire et couleur blanc nacré simulant une cire de bougie. La prise en charge est chirurgicale, sans oublier la substitution des déficits endocriniens hypophysaires dans cette forme de description.

## Patient et observation

**Figure 1:**
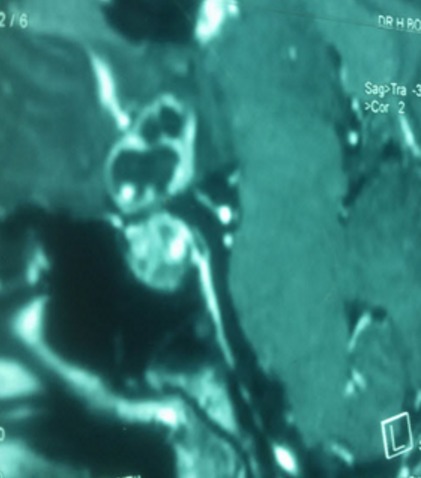
IRM hypothalamo-hypophysaire de notre patient: kyste épidermoide du patient intra et supra-sellaire, mesurant 18X15X27mm

Nous rapportons le cas du patient EY, âgé de 38 ans, mariée, père de 2enfants, sans antécédent pathologiques particuliers, notamment pas de notion de traumatisme crânien, qui a présenté une baisse de l'acuité visuelle gauche, des céphalées temporales gauches et des vomissements, avec des signes d'insuffisance antéhypophysaire des axe corticotrope, à type de pâleur, asthénie, des diarrhées liquidiennes, thyréotrope, à type de dépilation des avant bras, des jambes, des aisselles et pubienne, ralentissement et gonadotrope, à type de baisse de libido, des éjaculations et des érections matinales installés depuis 1an et chez qui l'IRM cérébrale a été en faveur d'un craniopharyngiome. Le bilan hormonal a objectivé un déficit corticotrope: cortisol: 2μg/dl, gonadotrope: Testostérone: 00 FSH: 2.31mu/mL, LH: 0.35Mui/ML, Prolactine: 10.86ng/ml, thyréotrope:T4libre: 2.95pmol/l, TSH: 3.03mUI/l. En effet l'IRM avait révélé un processus intra et supra-sellaire de signal hétérogène contenant des zones tissulaires hypo et isointenses en T1 et intermédiaire en T2 et hyperintenses en Flair, ne contenant pas de calcifications et des zones multiloculaires, à centre hyperintense en T1. Ce processus se rehausse par le gadolinium de manière modérée et hétérogène, nodulaire pour les composantes tissulaires et périphérique pour les composantes kystiques, mesurant 18*15*27mm de grands axes, comble la citerne suprasellaire, surélève le chiasma optique et les segments A1 des cérébrales antérieures et s'étend jusqu' à la région hypothalamique gauche. Par ailleurs, Il s'accompagne de plages hyperintenses en T2 et en Flair, hypointense en T1 et isointenses en diffusion, non réhaussées par le contraste, au niveau du tractus optique rétro-chiasmatique gauche (Figure 1). Le patient a été opéré par voie haute (abord temporal), l'examen anatomopathologique a montré un aspect morphologique compatible avec un kyste épidermoide. Les suites post-opératoires ont été marquées par l'apparition d'un diabète insipide pour lequel le patient a été mis sous minirin, les insuffisances gonadotrope et thyréotrope et corticotropes ont été substituées par un traitement hormonal. L'IRM de contrôle a montré une selle turcique vide. Le fond d'œil du post-opératoire a été en faveur de Pâleur papillaire bilatérale; que le patient avait auparavant, l'acuité visuelle s'est amélioré à 10/10 en bilatérale. Le chams visuel est resté altéré surtout à droite.

## Discussion

**Figure 2:**
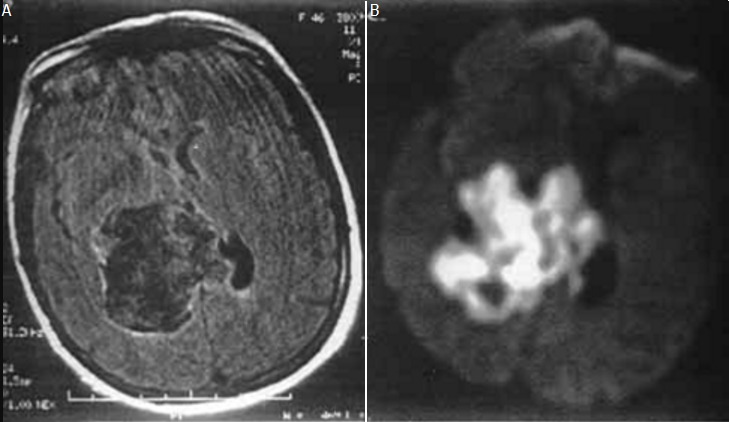
Aspect du kyste épidermoïde intraventriculaire: (A) en IRM (séquences FLAIR; (B) diffusion. La séquence FLAIR permet d’affirmer la nature « tissulaire » du kyste. Le kyste est hyperintense en séquence de diffusion. Ceci permet de clairement différencier le kyste épidermoïde du kyste arachnoïdien

Décrit depuis 1828 par Lepreste (Bensaid et al 1994), le kyste épidermoïde ou cholestéatome est une formation tumorale bénigne, d'origine dysembryoplasique résultant de la présence d'inclusion de tissu ectodermique lors de la fermeture du tube neural. Certains auteurs évoquent aussi la possibilité d'une origine accidentelle, à la suite d'introduction de fragments épidermiques dans l'espace sous-arachnoïdien après un traumatisme ouvert ou un acte chirurgical [1–3]. Il survient dans les espaces sous-arachnoïdiens; son siège de prédilection est la fosse cérébrale postérieure en particulier l'angle pontocérébelleux, la région sellaire et suprasellaire, la fosse temporale le diploé des os frontaux et pariétaux et rarement la région lombosacrée, les ventricules, les hémisphères cérébraux et le tronc cérébral [2]. L'extension se fait aussi dans les espaces sous-arachnoïdiens et c'est ainsi qu'un kyste de l'angle pontocérébelleux peut traverser l'incisure tentorielle et évoluer vers la région parasellaire [2, 3]. La TDM montre une masse de contours nets et irréguliers, « en carte géographique», de densité souvent hétérogène, proche de celle du liquide cérébro-spinal (LCS), sans prise de contraste [4, 5]. Les calcifications péri-capsulaires sont très rares. L'IRM en séquence pondérées en T1 et en T2 montre une lésion de signal proche de celui du LCS mais souvent hétérogène notamment en T2 en iso-ou hyposignal T1, hypersignal T2. Cet aspect plutôt liquidien et hétérogène du kyste épidermoïde, malgré son contenu riche en cholestérol, s'explique par la richesse en eau, en cellules desquamées et en kératine [4, 5]. Cette similitude de signal avec le liquide céphalorachidien pose parfois des problèmes de diagnostic différentiel avec le kyste arachnoïdien et certains kystes tumoraux [4, 5]. Cependant, certains arguments comme l'hétérogénéité du signal en T1, les contours nets et irréguliers ainsi que l'extension dans les espaces sous-arachnoïdiens plaident en faveur du kyste épidermoide, la séquence FLAIR montre un signal hétérogène très supérieur à celui du liquide céphalorachidien (Figure 2); en séquence CISS-3D, un signal légèrement moins intense que celui du liquide céphalorachidien; en diffusion, un signal hyperintense [4].

**Figure 3:**
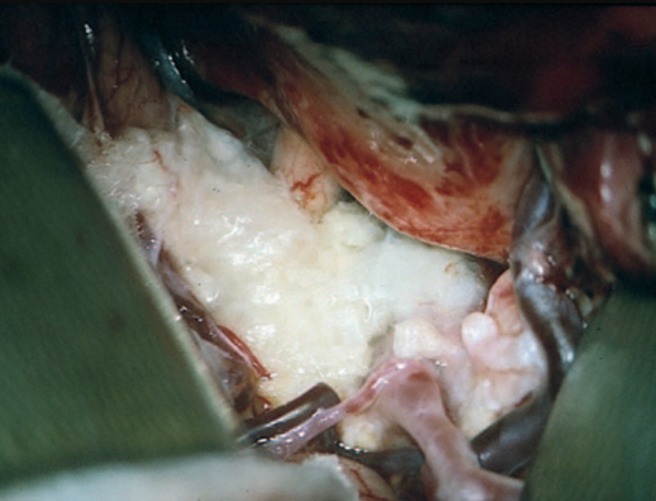
Vue peropératoire d’un kyste épidermoïde de l’angle pontocérébelleux (la tumeur perlée)

Par ailleurs, des kystes épidermoides hyperdenses ont été rapportés dans la littérature [5]. Chez nôtre patient, le signal hétérogène contenant des zones tissulaires hypo et isointenses en T1 et intermédiaire en T2 et hyperintenses en Flair, avec des plages hyperintenses en T2 et isointenses en diffusion, non rehaussées par le contraste et ne contenant pas de calcifications; tous ces arguments plaident en faveur du kyste épidermoide. Sur le plan histologique, l aspect macroscopique du kyste épidermoide est celui d une tumeur perlée avec une surface nodulaire et couleur blanc nacré simulant une cire de bougie [3, 4]. En microscopie, la paroi du kyste est fine et formée d'un épithélium pavimenteux stratifié avec des lamelles de desquamation. La croissance se fait à partir de la couche basale germinative de son épithélium et l'expansion par l'exfoliation lamellaire et l'accumulation de la kératine à l'intérieur de sa cavité, ce qui explique son évolution lente (Figure 3) [6]. Dans nôtre observation, vu que le patient n'a pas eu de traumatisme crânien, le kyste épidermoide serait congénital, augmentant progressivement de volume, ne devenant symptomatique qu'à la suite de l'envahissement de l'hypophyse, entrainant ainsi une insuffisance anté-hypophysaire globale (grande taille de 18*15*27mm). Le traitement des kystes épidermoïdes est toujours chirurgical, la voie d'abord est fronto-temporale pour les localisations sus-et péritentorielles et par un abord médian de la fosse postérieure en position assise pour les localisations sous-tentorielles [7]. La plupart des auteurs s'accordent à vouloir réaliser une exérèse complète afin de diminuer le risque de récidive mais aussi de méningite aseptique. Cela semble se vérifier puisqu'il semblerait que 50% des exérèses rapportées dans la littérature soient complètes. Ces récidives s'échelonnent entre quelques mois et 21 ans [7]. La morbi-mortalité post-opératoire des kystes épidermoïdes demeure importante, des complications neurologiques sont fréquentes a type de troubles mnésiques, atteinte des paires crâniennes définitive, dysphasie, syndrome cérébelleux, hémiparésie, coma [7–9].

## Conclusion

Le kyste épidermoïde est une tumeur bénigne d'évolution linéaire lente mais inéluctable, justifiant un traitement chirurgical. Le diagnostic devient de plus en plus aisé, notamment avec l'avènement de séquences de diffusion en IRM. Cette observation plaide en faveur d l'origine congénitale et l'évolution lente de ce type de tumeurs.

## Conflits d’intérêts

Les auteurs ne déclarent aucun conflit d'intérêts.
